# Genome analysis provides insights into the epidemiology of infection with *Flavobacterium psychrophilum* among farmed salmonid fish in Sweden

**DOI:** 10.1099/mgen.0.000241

**Published:** 2018-12-13

**Authors:** Robert Söderlund, Mikhayil Hakhverdyan, Anna Aspán, Eva Jansson

**Affiliations:** ^1^​ Department of Microbiology, National Veterinary Institute (SVA), 75189, Uppsala, Sweden; ^2^​ Department of Animal Health and Antimicrobial Strategies, National Veterinary Institute (SVA), Uppsala, Sweden

**Keywords:** *Flavobacterium psychrophilum*, genomics, aquaculture, molecular epidemiology, antimicrobial resistance, rainbow trout

## Abstract

The pathogen *Flavobacterium psychrophilum* is a major problem for the expanding salmonid fish farming industry in Sweden as well as worldwide. A better understanding of the phylogeography and infection routes of *F. psychrophilum* outbreaks could help to improve aquaculture profitability and the welfare of farmed fish while reducing the need for antibiotics. In the present study, high-throughput genome sequencing was applied to a collection of *F. psychrophilum* isolates (*n*=38) from outbreaks on fish farms in different regions of Sweden between 1988 and 2016. Antibiotic susceptibility tests were applied to a subset of the isolates and the results correlated to the presence of genetic resistance markers. We show that *F. psychrophilum* clones are not regionally biased and that new clones with a higher degree of antibiotic resistance have emerged nationwide during the study period. This supports previous theories of the importance of live fish and egg trade as a route of infection. Continuous monitoring of recovered isolates by high-throughput sequencing techniques in the future could facilitate tracing of clones within and between countries, as well as the detection of emergent virulent or antibiotic-resistant clones. This article contains data hosted by Microreact.

## Data Summary

Illumina MiSeq whole genome sequencing data generated from 38 Swedish isolates and three Finnish isolates of *Flavobacterium psychrophilum* are available as 2×250 bp paired-end reads and draft assemblies from the European nucleotide archive (ENA), https://www.ebi.ac.uk/ena, under project accession number PRJEB27605 and at figshare: https://figshare.com/s/ab8cb75bba658aeb6968 Further data including information regarding the origin of isolates, genomic typing results and results from phenotypic antibiotic resistance testing are presented in Tables S1 and S2 (available in the online version of this article).

**Impact Statement**

Comparing the complete genetic material, the genome, between strains of bacteria has rapidly emerged as a standard method that has revolutionized many aspects of infectious disease control, both in public health and in veterinary medicine. In the present project we have studied the genomes of the fish pathogen *Flavobacterium psychrophilum*, recovered from rainbow trout and other salmonids during disease outbreaks on Swedish fish farms between 1988 and 2016. By comparing the genomes, we show that new variants of the bacteria have appeared over the last decade, partially replacing the original variants over large geographical distances. The new variants of the bacteria are more resistant to antibiotics compared to those found earlier in the study period. This widespread emergence of new variants points towards trade of live fish and fish eggs as the major long-term source of *Flavobacterium* outbreaks rather than local or environmental sources of infection. Taking this into account, control measures can be put in place to reduce the number of outbreaks in the future, improving animal welfare while reducing the use of antibiotics and economic losses for the growing fish farming industry in Sweden.

## Introduction

Fish farming is a rapidly growing and profitable industry in Sweden. Salmonid fish dominate the production output; the most common species farmed is rainbow trout (*Oncorhynchus mykiss*), but smaller volumes of Arctic charr (*Salvelinus alpinus*), brown trout (*Salmo trutta*) and Atlantic salmon (*Salmo salar*) are also produced [1]. Salmonid fish are raised for consumption as well as for stocking of lakes and rivers to improve recreational fishing and to replenish populations hindered from migration by hydroelectric dams. Fish for food production are predominantly raised in cage systems in natural freshwater lakes. Although these systems are a cost-effective way to provide hygienic conditions for the farmed fish, they allow wild and farmed fish to exchange pathogens and serve as reservoirs of infectious disease agents for each other. The health status of Swedish farmed fish is generally good, but outbreaks of infectious diseases occur regularly and cause major economic losses. One of the main pathogens that cause economic losses for salmonid fish farms in Sweden, as well as worldwide, is the bacterium *Flavobacterium psychrophilum*, the causative agent of diseases referred to as bacterial cold-water disease (BCWD) and rainbow trout fry syndrome (RTFS) [1, 2]. Infection can cause high mortality in young fish but also causes morbidity in adult fish. Treatment of infection by *F. psychrophilum* together with the related bacterium *Flavobacterium columnare* accounts for an estimated 50–60 % of the antibiotics used in Swedish aquaculture [2]. A better understanding of the routes of infection for farms affected by *F. psychrophilum* could therefore allow more effective preventative measures to improve aquaculture profitability and animal welfare, while reducing antibiotic usage.

In recent years high-throughput sequencing methods have been established as the new gold standard for characterization of bacterial pathogens, both for research purposes and for outbreak investigation and tracing of sources of infection. Previous studies using such whole-genome sequencing techniques have provided new insights into the evolution, global diversity and pathogenesis mechanisms of *F. psychrophilum* [3–5]. In the present study, we have applied high-throughput sequencing-based methodologies to a collection of *F. psychrophilum* from fish farms in different regions of Sweden between 1988 and 2016 in order to better understand the population structure and phylogeography of the pathogen over time.

## Methods

### Isolates

Representative isolates (*n*=38) from samples collected at 26 fish farms in 14 counties in Sweden were included in the study. Three isolates from fish farms in Finland were also included for reference. Included isolates were verified as *F. psychrophilum* by MALDI-TOF MS performed on a MALDI Biotyper System (Bruker) by use of main spectra projections prepared and validated for identification of *F. psychrophilum* and *F. columnare* according to Bruker’s instructions. Fish farm locations were aggregated to county level to protect the anonymity of study participants. The isolates were collected in connection with disease outbreaks between 1988 and 2016 and originated from rainbow trout (*n*=36), Arctic charr (*n*=2), brown trout (*n*=1) and fish farm water samples (*n*=2). Of the fish isolates, 33 were from kidneys, one each was from skin, fin, gill and abscess samples while the remaining were from unspecified tissues (see Table S1 for details).

### Phenotypic antibiotic resistance testing

Antibiotic susceptibility was analysed at 20 °C by using a broth microdilution technique, adapted for aquatic bacteria according to recommendations from the Clinical and Laboratory Standards Institute [6], by use of microdilution panels (VetMic Aquatic; SVA) and the control strain *Aeromonas salmonicida* subsp. *salmonicida* ATCC 33658. The minimum inhibitory concentration (MIC) was recorded as the lowest concentration of the antibiotics that inhibited bacterial growth. Epidemiological cut-offs for florfenicol, oxolinic acid and oxytetracyklin were set according to Smith *et al*. [7].

### Genome analysis

DNA from colony material was extracted using an EZ1 Biorobot system (Qiagen). Libraries were prepared using the Nextera XT DNA Sample Preparation Kit (Illumina). Fragment sizes were verified to be in the range 250–1000 bp and molar concentrations were determined using an Agilent 2100 Bioanalyzer and Agilent High Sensitivity DNA Kit (Agilent Technologies). Sequencing was performed on a MiSeq instrument (Illumina) as paired-end 2×250 bp reads. Sequence data were uploaded to the European Nucleotide Archive (ENA) and are available under project accession number PRJEB27605. Sequence data were subsampled for further analysis so as not to exceed approximately 100× coverage based on the expected genome size; based on the draft genome sizes produced, the coverage range was 73×–97× for the included isolates. SNP typing was performed by mapping reads to the complete *F. psychrophilum* JIP02/86 genome sequence [4] using Bowtie 2 2.2.7 [8] with default settings and expected fragment lengths of 0–1000 bp, and calling SNPs with SAMtools 1.3.1 [9] using standard settings with the exceptions of excluding indels and setting ploidy to 1. SNPs were filtered, requiring a single variant allele for each included variable site, an overall quality >100 for each variable site, and for each variable site to be present and have a quality >25 in all isolates. Furthermore, sites with a conflicting genotype call with a quality of >10 % of the primary call quality were excluded. SNP variation between isolates was visualized using the NeighborNet algorithm in SplitsTree 4.14.4 [10]. A representative of each SNP cluster was verified as *F. psychrophilum* by the Centre for Genomic Epidemiology (CGE) SpeciesFinder 16S rRNA-based tool [11]. Draft genome assemblies were created using SPAdes 3.5.0 [12] with the '-careful' option active. Assembly metrics were evaluated using quast 2.3 [13]. Assemblies were uploaded to ENA under the same project accession number as the read data. Multi-locus sequence typing [14, 15] (MLST) was performed on draft assemblies, as implemented in the Centre for Genomic Epidemiology MLST web service (https://cge.cbs.dtu.dk/services/MLST). New allele combinations were submitted to PubMLST (https://pubmlst.org) for sequence type (ST) assignment. Variation in the DNA gyrase A subunit gene (*gyrA*) was investigated by extracting the sequence of the quinolone resistance-determining region [16] from each draft assembly using blast+. The presence or absence of possible tetracycline resistance genes previously reported in *Flavobacterium* (*tetA* GenBank X00006, *tetM* X56353.1 [17]; *tetX* AM398681.2 [4]) was investigated by blast+ analysis requiring >75 % sequence similarity and a minimum hit length of 50 bp.

## Results

### Genome analysis

**Fig. 1.**
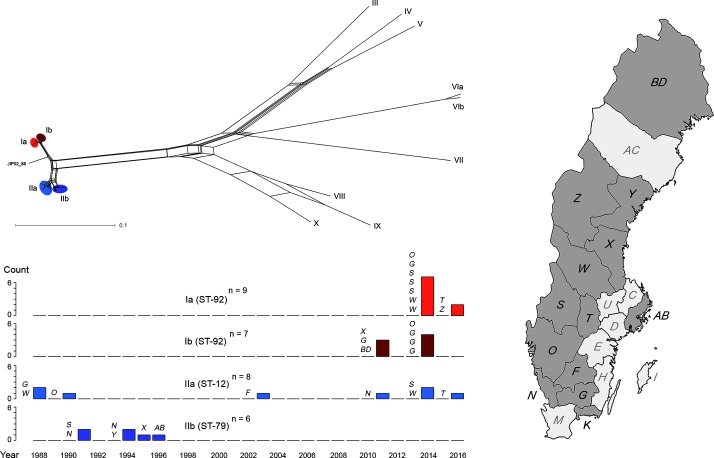
Population structure and spatio-temporal distribution of lineages of *F. psychrophilum* among Swedish farmed salmonid fish. Upper: NeighborNet representation of the genetic relationship between all included isolates and the reference genome JIP02/86 based on whole-genome SNP typing, annotated with SNP cluster designation and with the four major clusters highlighted. Lower: number of observations of the four major SNP clusters per year between 1988 and 2016. Each bar is annotated with county codes (in italics) showing the locations of observations of a given cluster in a given year. Right: map of Sweden showing county borders and county codes. Counties of origin of one or more isolates in the study are highlighted in darker grey.

Draft genome assemblies of the included isolates were in the size range 2.4–2.9 Mbp, with a G+C content of 32.4–32.8 % (see Table S1 for full assembly metrics), which is close to values from complete genomes [4, 18]. All isolates were confirmed as representing *F. psychrophilum* based on 16S rRNA typing. The results from whole-genome SNP typing were consistent with MLST for all isolates, with SNP clusters Ia/Ib corresponding to ST-92, and clusters IIa, IIb and VIa/VIb corresponding to ST-12, ST-79 and ST-23 respectively (Fig. 1). Most isolates belonged to the large clonal complex 10 (CC-ST10) as defined by Nilsen *et al*. [19] (Table S2). The newly assigned ST-337 was consistent with an allocation to CC-ST236, while the newly assigned ST-338 differed by a single locus each from ST-132 and ST-237 in the PubMLST database, creating a new CC (CC-ST338). Isolates with unique STs in the study did not cluster with any other isolates in terms of SNP variation. As expected, SNP typing was far more discriminatory than MLST, separating each ST into several subgroups and providing a unique profile for all isolates in the study except for SE568 and SE569, which were from the same outbreak. Reticulation was present in the NeighborNet network, consistent with previous observations of recombination in *F. psychrophilum* [19] (Fig. 1). No clear patterns of geographical distribution for genotypes were observed, but certain genotypes were restricted in time with the same SNP clusters occurring on several fish farms during limited periods.

### Phenotypic and genotypic antibiotic resistance testing

Isolates in SNP clusters IIa and IIb (corresponding to ST-12 and ST-79) were generally sensitive to florfenicol, oxolinic acid and oxytetracycline, while isolates in clusters Ia and Ib (ST-92) were sensitive to florfenicol but resistant to the other two antibiotics. An amino acid substitution mutation in the quinolone resistance-determining region of the *gyrA* gene (Thr83-Ile) was observed in all ST-92 isolates, but absent in ST-12 and ST-79, providing an explanation for the difference in oxolinic acid resistance [16]. Two isolates in SNP cluster IIb had other amino acid substitutions at the same position (*gyrA* Thr83-Arg and Thr83-Ala). The only putative tetracycline resistance gene detected was *tetX*, which occurred in all ST-12 and ST-79 isolates and in 14 of 17 ST-92 isolates but absent in all other STs. The rare and unique genotypes analysed had variable resistance profiles, but all were sensitive to florfenicol. Full results are presented in Table S2.

## Discussion

*F. psychrophilum* is currently endemic in Sweden and has been shown to colonize various wild salmonid and non-salmonid fish species, with or without causing signs of disease [2]. The bacteria have also been shown to be capable of surviving for at least 300 days in freshwater, as well as being aided in their survival by the presence of sediment [20]. This suggests that farmed fish are at risk of continuous environmental exposure, with outbreaks perhaps occurring when fish are weakened by stress or when immunologically naïve young fish are exposed to the pathogen. However, *F. psychrophilum* is also suspected of being capable of vertical transmission within salmonid eggs [21]. Based on the appearance of the disease following import of eggs and the detection of *F. psychrophilum* in imported eggs, as well as the occurrence of the same clones in multiple countries, this route of infection has historically been considered important [19, 22, 23]. The present data from Swedish farmed salmonid fish support this suggestion, as unrelated lineages of the bacteria dominated during the periods 1988–1997 (ST-79) and 2011–2016 (ST-92). The emergence of the antibiotic-resistant ST-92 lineage at fish farms in several counties throughout the country over the course of a decade strongly suggests a common, external source of infection, probably the import of eggs or fish from one or several shared suppliers. While we know such imports occur, unfortunately no detailed records are available for the present isolates. However, we note that whole genome sequencing is well suited to monitor such introduction events and to trace spread of infection between countries with better resolution compared to MLST and other older methods. Such international comparisons will be facilitated by the publication of the presented sequence data. Future studies could also use these techniques to determine if newly introduced variants of *F. psychrophilum* are transmitted to wild fish in areas around fish farms. ST-12 was present among Swedish salmonid fish throughout the study period, with observations ranging from 1988 to 2016. ST-12 has been isolated from salmonid fish in several countries worldwide (https://pubmlst.org) and belongs to a CC associated with outbreaks in several Nordic countries [19]. It may thus represent an earlier global expansion of bacteria from shared sources via live animal trade.

Of the 38 isolates from Sweden included in the present study, 17 have previously been subjected to MLST by traditional Sanger sequencing [19]. For nine of these isolates, results from whole genome sequencing and Sanger data gave complete matches in MLST profiles, but for eight isolates differences were found (Table S2). This discrepancy was only found in the *tuf* gene sequences. This could be due to a mix of closely related clones in the frozen bacterial stocks representing variation in the source outbreak, as subcultivation is necessary before DNA extraction, and different DNA preparations were used for the earlier Sanger sequencing and the present whole genome sequencing. It is notable that the differences were found only in one of the seven housekeeping genes, i.e. *tuf*, which might indicate a lower stability in this gene, and that care should be taken when judging isolates as different by variation in this gene only. Sequencing of multiple isolates from the same source should be a priority in the future and will improve our understanding of within-outbreak variation.

It is notable that most isolates for the period 1988–2003 in the present study belong to the quinolone- and oxytetracycline-sensitive lineages of the bacterium, while the more recent isolates predominantly belong to quinolone- and oxytetracycline-resistant lineages. Our genome data show that *gyrA*-mediated quinolone resistance has evolved or been acquired independently multiple times among the strains recovered in the study. In contrast, we could find no obvious genetic basis for the observed tetracycline resistance in these more recent isolates. We found the presence or absence of the *tetX* gene to be unrelated to phenotypic tetracycline resistance as the gene was present in multiple sensitive isolates and absent from resistant isolates. This is in concordance with similar observations made in *Flavobacterium spartansii* [24]. Continuous surveillance of antibiotic resistance of clinical isolates of fish pathogens in Sweden in the SWEDRES/SWARM programme has revealed a marked increase in resistance to tetracycline and quinolones in *F. psychrophilum* since 2005, despite limited use of these substances for therapeutic use in fish farms. The increase is therefore unlikely to be the result of a selective pressure from domestic antibiotic use, but rather introduction via fish or fish products followed by dissemination within the fish farming industry.

In conclusion, by analysis of a temporally and geographically representative set of *F. psychrophilum* isolates from outbreaks among Swedish farmed salmonid fish, we show repeated nationwide introductions of new clones. This highlights the need for improved microbiological security for eggs and fry in the fish farming supply chain. Continuous monitoring of recovered isolates by high-throughput sequencing techniques in the future could facilitate tracing of clones within and between countries, as well as early detection of the emergence of new virulent or antibiotic-resistant clones to guide the selection of strains for vaccine development. Genomic techniques could also help to improve our understanding of the infection interplay between farmed and wild fish populations.

## Data bibliography

1. Complete genome sequence of the JIP02/86 strain of F. psychrophilum, available from GenBank (https://www.ncbi.nlm.nih.gov/nuccore/AM398681.2), from Duchaud E, Boussaha M, Loux V, Bernardet JF, Michel C, Kerouault B, *et al*. Complete genome sequence of the fish pathogen Flavobacterium psychrophilum. Nat Biotechnol. 2007;25(7):763-9.
2. The F. psychrophilum MLST database hosted by the University of Oxford (https://pubmlst.org/fpsychrophilum), Keith A. Jolley, James E. Bray, and Martin C. J. Maiden, Open-access bacterial population genomics: BIGSdb software, the PubMLST.org website and their applications. Wellcome Open Res. 2018; 3: 124.
3. Reference sequence for the tetA gene from GenBank X00006 (https://www.ncbi.nlm.nih.gov/nuccore/X00006), originally from Waters, S.H., Rogowsky, P., Grinsted, J., Altenbuchner, J. and Schmitt,R. The tetracycline resistance determinants of RP1 and Tn1721: nucleotide sequence analysis. Nucleic Acids Res. 11 (17), 6089-6105 (1983)
4. Reference sequence for the tetM gene from GenBank X56353.1 (https://www.ncbi.nlm.nih.gov/nuccore/X56353.1), originally from Burdett,V, Nucleotide sequence of the tet(M) gene of Tn916. Nucleic Acids Res. 18 (20), 6137 (1990)
5. Reference sequence for the tetX gene from the JIP02/86 genome (see 1.).
6. Clonal complex assignments based on MLST data from Nilsen H, Sundell K, Duchaud E, Nicolas P, Dalsgaard I, Madsen L, *et al*. Multilocus sequence typing identifies epidemic clones of Flavobacterium psychrophilum in Nordic countries. Applied and environmental microbiology. 2014;80(9):2728-36.

## Supplementary Data

Supplementary File 1
