# Supplementary material for: Genome analysis provides insights into the epidemiology of infection with Flavobacterium psychrophilum among farmed salmonid fish in Sweden
Source: Microb Genom. 2018 Dec 13;4(12):e000241. doi: 10.1099/mgen.0.000241 (PMC6412038; doi:10.1099/mgen.0.000241)
Supplement: Supplementary File 1 [file mgen-4-241-s001.pdf]

Supplementary table 1: Origin of isolates and sequence data metrics

ID	Origin				Sequencing and assembly metrics			
ID	County	Year	Host	Sample	Est. coverage	Assembly (Mbp)	N50 (kbp)	Contig count
SE476	N	2011	Rainbow trout	kidney	79x	2.61	61.4	122
SE552	S	2014	Rainbow trout	kidney	91x	2.74	85.3	98
SE553	W	2014	Rainbow trout	kidney	95x	2.71	94.3	70
SE010	G	1988	Rainbow trout	unknown	80x	2.81	104	105
SE009	W	1988	Rainbow trout	kidney	81x	2.73	107	98
SE022	O	1990	Rainbow trout	kidney	79x	2.67	62.4	116
SE409	F	2003	Rainbow trout	kidney	80x	2.75	104	107
SE554	T	2016	Rainbow trout	kidney	92x	2.81	137	91
SE026	S	1991	Rainbow trout	skin	79x	2.75	117	100
SE028	N	1991	Rainbow trout	kidney	80x	2.75	114	111
SE080	N	1994	Rainbow trout	kidney	79x	2.72	125	94
SE091	Y	1994	Rainbow trout	kidney	86x	2.73	66.7	106
SE117	X	1995	Rainbow trout	kidney	78x	2.79	104	109
SE164	AB	1996	Rainbow trout	kidney	86x	2.76	137	97
SE555	O	2014	Rainbow trout	kidney	79x	2.66	55.8	131
SE556	G	2014	Rainbow trout	kidney	93x	2.76	102	98
SE557	S	2014	Rainbow trout	kidney	78x	2.75	97.4	112
SE558	S	2014	Rainbow trout	kidney	90x	2.77	56.9	135
SE559	S	2014	Rainbow trout	kidney	90x	2.79	92.3	124
SE560	W	2014	Rainbow trout	kidney	93x	2.76	102	110
SE561	W	2014	Rainbow trout	kidney	92x	2.74	80.4	124
SE562	Z	2016	Rainbow trout	abscess	90x	2.81	65.0	124
P18-2/11	NA/Finland	2011	Rainbow trout	kidney	91x	2.73	45.7	146
SE563	T	2016	Rainbow trout	kidney	92x	2.77	97.5	120
SE564	G	2014	Rainbow trout	kidney	81x	2.57	43.7	143
SE565	O	2014	Rainbow trout	kidney	88x	2.39	57.8	120
SE566	G	2014	Rainbow trout	kidney	85x	2.81	99.2	104
SE567	G	2014	Rainbow trout	kidney	93x	2.78	102	111
SE477	G	2011	Rainbow trout	kidney	81x	2.74	77.2	133
SE482	BD	2011	Rainbow trout	kidney	80x	2.64	95.0	146
SE483	X	2011	Rainbow trout	kidney	76x	2.74	90.4	129
SE568	BD	2014	Arctic char	kidney	96x	2.64	65.2	90
SE569	BD	2014	Arctic char	gill	87x	2.75	95.2	101
M2/99	NA/Finland	1999	NA	water	96x	2.68	66.3	99
SE328	K	2001	Rainbow trout	kidney	84x	2.70	165	103
SE474	G	2011	Rainbow trout	kidney	82x	2.69	136	121
SE570	BD	2016	Rainbow trout	kidney	97x	2.69	89.6	112
H7/00	NA/Finland	2000	NA	water	85x	2.76	98.0	136
SE571	O	2014	Rainbow trout	kidney	80x	2.90	80.0	183
SE027	S	1991	Rainbow trout	unknown	73x	2.86	58.8	274
SE055	X	1993	Brown trout	fin	80x	2.40	70.2	161

Supplementary table 2: Molecular and antibiotic resistance data

ID	Molecular typing					Antibiotic resistance					Previously observed***	
ID	SpeciesFinder 16S	MLST	ST	CC	SNP cluster	Florfenicol	Oxolinic acid	*gyrA*	Oxytetracycline	*tetX*	*tuf* allele	MLST
SE476	*F. psychrophilum*	2-2-2-8-2-2-7	12	CC-ST10*	IIa	NT	NT	WT	NT	Pos	NA	NA
SE552	NT	2-2-2-8-2-2-7	12	CC-ST10*	IIa	S	S	WT	S	Pos	NA	NA
SE553	NT	2-2-2-8-2-2-7	12	CC-ST10*	IIa	S	S	WT	S	Pos	NA	NA
SE010	NT	2-2-2-8-2-2-7	12	CC-ST10*	IIa	NT	NT	WT	NT	Pos	NA	NA
SE009	NT	2-2-2-8-2-2-7	12	CC-ST10*	IIa	NT	NT	WT	NT	Pos	NA	NA
SE022	NT	2-2-2-8-2-2-7	12	CC-ST10*	IIa	NT	NT	WT	NT	Pos	NA	NA
SE409	NT	2-2-2-8-2-2-7	12	CC-ST10*	IIa	NT	NT	WT	NT	Pos	2	10
SE554	NT	2-2-2-8-2-2-7	12	CC-ST10*	IIa	S	S	WT	S	Pos	NA	NA
SE026	*F. psychrophilum*	2-8-2-8-2-2-2	79	CC-ST10*	IIb	NT	NT	WT	NT	Pos	NA	NA
SE028	NT	2-8-2-8-2-2-2	79	CC-ST10*	IIb	NT	NT	WT	NT	Pos	NA	NA
SE080	NT	2-8-2-8-2-2-2	79	CC-ST10*	IIb	NT	NT	WT	NT	Pos	3	235
SE091	NT	2-8-2-8-2-2-2	79	CC-ST10*	IIb	NT	NT	Thr83-Arg	NT	Pos	49	238
SE117	NT	2-8-2-8-2-2-2	79	CC-ST10*	IIb	NT	NT	WT	NT	Pos	7	240
SE164	NT	2-8-2-8-2-2-2	79	CC-ST10*	IIb	NT	NT	Thr83-Ala	NT	Pos	7	240
SE555	*F. psychrophilum*	2-2-2-2-2-3-41	92	CC-ST10*	Ia	S	R	Thr83-Ile	R	Neg	NA	NA
SE556	NT	2-2-2-2-2-3-41	92	CC-ST10*	Ia	S	R	Thr83-Ile	R	Pos	NA	NA
SE557	NT	2-2-2-2-2-3-41	92	CC-ST10*	Ia	S	R	Thr83-Ile	R	Pos	NA	NA
SE558	NT	2-2-2-2-2-3-41	92	CC-ST10*	Ia	S	R	Thr83-Ile	R	Pos	NA	NA
SE559	NT	2-2-2-2-2-3-41	92	CC-ST10*	Ia	S	R	Thr83-Ile	R	Pos	NA	NA
SE560	NT	2-2-2-2-2-3-41	92	CC-ST10*	Ia	S	R	Thr83-Ile	R	Pos	NA	NA
SE561	NT	2-2-2-2-2-3-41	92	CC-ST10*	Ia	S	R	Thr83-Ile	R	Pos	NA	NA
SE562	NT	2-2-2-2-2-3-41	92	CC-ST10*	Ia	S	R	Thr83-Ile	R	Pos	NA	NA
P18-2/11	NT	2-2-2-2-2-3-41	92	CC-ST10*	Ia	NT	NT	Thr83-Ile	NT	Pos	NA	NA
SE563	NT	2-2-2-2-2-3-41	92	CC-ST10*	Ia	S	R	Thr83-Ile	R	Pos	NA	NA
SE564	*F. psychrophilum*	2-2-2-2-2-3-41	92	CC-ST10*	Ib	S	R	Thr83-Ile	R	Pos	NA	NA
SE565	NT	2-2-2-2-2-3-41	92	CC-ST10*	Ib	S	S	Thr83-Ile	R	Neg	NA	NA
SE566	NT	2-2-2-2-2-3-41	92	CC-ST10*	Ib	S	R	Thr83-Ile	R	Pos	NA	NA
SE567	NT	2-2-2-2-2-3-41	92	CC-ST10*	Ib	S	R	Thr83-Ile	R	Pos	NA	NA
SE477	NT	2-2-2-2-2-3-41	92	CC-ST10*	Ib	NT	NT	Thr83-Ile	NT	Pos	NA	NA
SE482	NT	2-2-2-2-2-3-41	92	CC-ST10*	Ib	NT	NT	Thr83-Ile	NT	Neg	NA	NA
SE483	NT	2-2-2-2-2-3-41	92	CC-ST10*	Ib	NT	NT	Thr83-Ile	NT	Pos	NA	NA
SE568	*F. psychrophilum*	12-11-7-13-9-9-13	23	NA	VI	S	R	WT	S	Neg	NA	NA
SE569	NT	12-11-7-13-9-9-13	23	NA	VI	S	R	WT	R	Neg	NA	NA
M2/99	NT	12-11-7-13-9-9-13	23	NA	VI	NT	NT	WT	NT	Neg	NA	NA
SE328	*F. psychrophilum*	1-15-14-13-1-2-7	124	CC-ST124*	IX	NT	NT	WT	NT	Neg	2	241
SE474	*F. psychrophilum*	1-15-16-13-1-2-7	126	CC-ST124*	VIII	NT	NT	WT	NT	Neg	NA	NA
SE570	*F. psychrophilum*	2-15-2-13-2-1-7	125	CC-ST125*	X	S	S	WT	R	Neg	NA	NA
H7/00	*F. psychrophilum*	2-8-5-37-2-33-26	133	NA	VII	NT	NT	WT	NT	Neg	NA	NA
SE571	*F. psychrophilum*	2-22-3-3-3-4-3	181	CC-ST191*	IV	S	R	WT	R	Neg	NA	NA
SE027	*F. psychrophilum*	3-22-3-8-3-8-3	337	CC-ST236*	V	NT	NT	Thr83-Ala	NT	Neg	2	234
SE055	*F. psychrophilum*	3-2-11-49-44-11-3	338	CC-ST338**	III	NT	NT	WT	NT	Neg	2	237

NT = not typed

*From Nilsen et al 2014
**New CC
NA = No CC defined

NT = not typed
R = resistant, S = sensitive

WT = wild type

***MLST variation observed in same sample compared to a previous study (see main text)
